# Observed and simulated hydro-climatic data for the lake Chad basin, Africa

**DOI:** 10.1016/j.dib.2019.104043

**Published:** 2019-05-23

**Authors:** Rashid Mahmood, Shaofeng Jia

**Affiliations:** a Key Laboratory of Water Cycle and Related Land Surface Processes/Institute of Geographic Science and Natural Resources Research, Chinese Academy of Sciences, Beijing, 100101, China; b Qinghai Key Laboratory of Basin Water Cycle and Ecology, Qinghai Institute of Water Resources and Hydropower, Xining, China; c School of Geographical Sciences, Qinghai Normal University, Xining, China

**Keywords:** The Lake Chad basin, Observed climate data, Observed streamflow, Natural streamflow, The CRU climatic data, Daily generated climate data

## Abstract

Lake Chad is one of the largest lakes in the world, but extremely vulnerable to the changing climate and human activities in the basin. The Lake Chad basin is one of the largest endorheic basins in the world and straddles the borders of Central African Republic, Chad, Libya, Niger, Nigeria, Algeria, Cameroon, and Sudan. In the last 40–50 years, the lake has shrunk from a surface area of 25,000 km^2^ to 2000 km^2^. However, the availability and quality of hydro-climatic data for researchers are major barriers to research. Since observed station data is highly sparse in the basin and difficult to collect, monthly climatic data was extracted from the gridded Climate Research Unit (CRU) dataset. The gridded CRU temperature and rainfall data was extracted at 81 points, and monthly temperature and rainfall data was converted into daily data for hydrologic modelling in Mahmood and Jia [1]. This data article also includes observed streamflow data of 3 hydrometric stations and rainfall data of 11 stations, which was obtained from the Lake Chad Basin Commission. Natural streamflow data simulated with hydrologic model at N'Djamena station on the Chari-Logone River is also included in this data article.

**Table:** Specifications table

Subject area	*Environment*
More specific subject area	*Climate Change, Water Resources assessment and management*
Type of data	*Tables (Time series data in Excel)*
How data was acquired	*Observed monthly streamflow and rainfall data was obtained from the LCBC. Monthly maximum temperature, minimum temperature, mean temperature, and rainfall were extracted from CRU gridded dataset. Monthly CRU climatic data was converted into daily by Monthly to Daily Weather Converter (MODAWEC). Daily natural streamflow was generated by HEC-HMS (Hydrologic Modelling System) using daily converted CRU climatic data.*
Data format	*Raw data (Monthly raw streamflow and rainfall), Generated (daily generated temperature and rainfall), Simulated (daily simulated natural streamflow)*
Experimental factors	*Climate Data Operator (CDO), MODAWEC, HEC-geoHMS, HEC-HMS were used to compile the dataset*
Experimental features	*Monthly climatic data was extracted from CRU datasets using CDO. The extracted data was evaluated with the observed datasets and converted into daily climatic data for hydrologic modelling using MODAWEC. Daily natural streamflow was generated by HEC-HMS for water resources management.*
Data source location	*The Lake Chad basin, Africa*
Data accessibility	*Data is available with this article*
Related research article	*Mahmood R, Jia S. Assessment of hydro-climatic trends and causes of dramatically declining stream flow to Lake Chad, Africa, using a hydrological approach. Science of The Total Environment, 2019.*https://doi.org/10.1016/j.scitotenv.2019.04.219. [1]

**Table:** 

**Value of the Data** • This data can be used in different climatic studies such as to explore hydro-climatic trends, climate variability, and climatic changes, and hydrologic studies in the Lake Chad basin (LCB). • Since most hydrologic models require daily climatic data, the generated daily climatic dataset can substantially reduce the time of researchers working on the LCB, where only monthly climatic data is available. • The daily generated data can also be used in the statistical downscaling of coarse resolution Global Climatic Models. • The simulated natural streamflow data can be very helpful for researchers working on water resources management and planning in the LCB.

## 1 Data

**Fig. 1:**
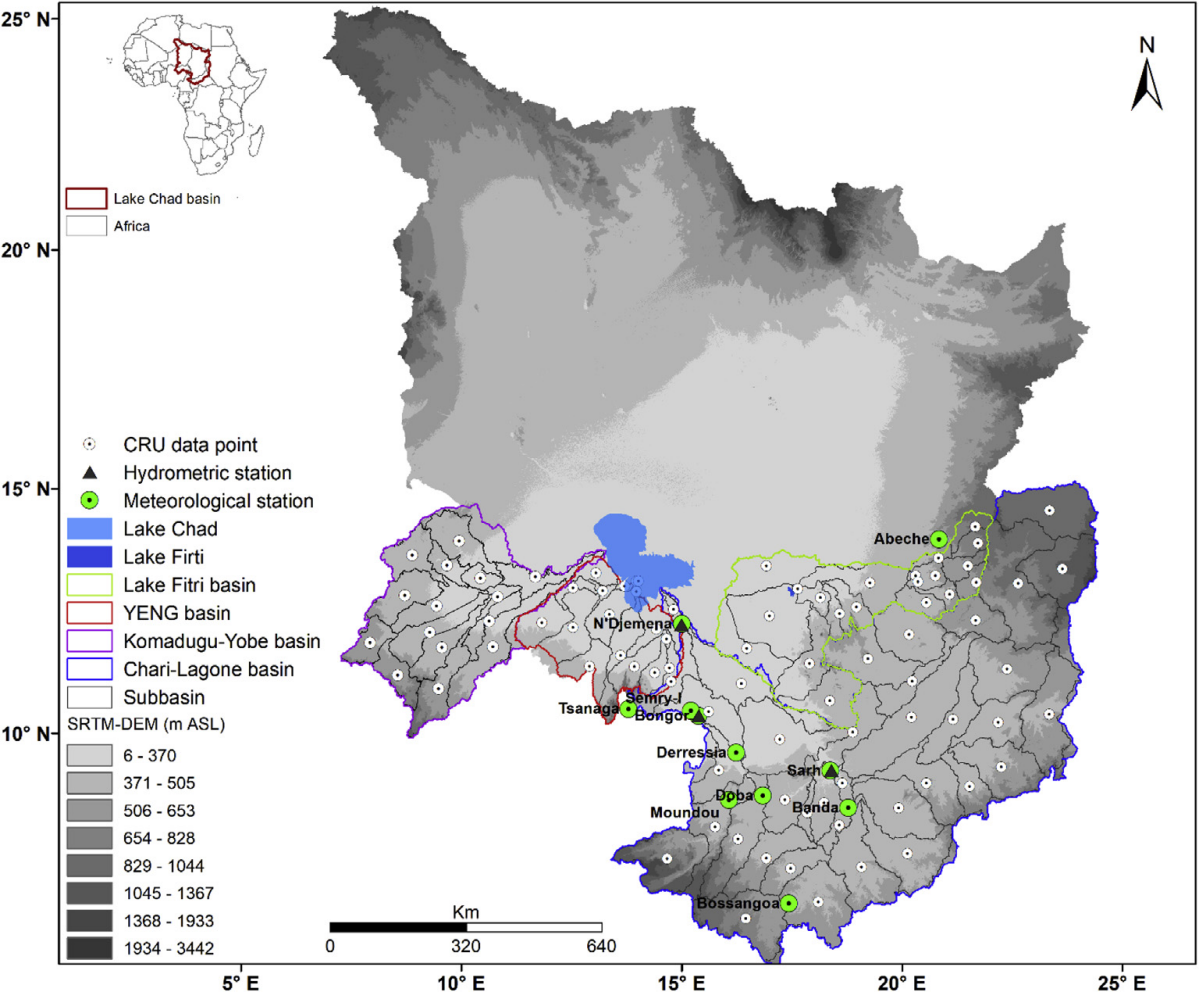
Locations of observed hydro-climatic stations and CRU data points (the points where data was extracted from gridded CRU data) in the main river basins of the LCB. YENG refers to the Yedseram, El-beid, Ngadda, and Gubio River basins.

Fig. 1 shows the location of the major river basins (i.e., the Chari-Logone, Komadugu-Yobe, Gubio, Ngadda, Yedseram, El-beid, and Lake Fitri basins) in the LCB, which directly or indirectly contribute water to Lake Chad, and the locations of observed hydro-climatic stations as well as the points at which CRU data was extracted. Some basic information (e.g., data period and elevation) about 11 rainfall stations and 3 streamflow gauges is described in Table 1. The comprehensive description of datasets included in the present data article are given in Table 2, which gives information about data folders, data files (Excel format), number of data files in each folder, data variables, and data period of each dataset. Each data file (Excel) contains basic information of data (e.g., latitude, longitude, elevation, station or the names of extracted data points) and time series data.

**Table 1:** Basic information of hydro-climatic gauges in the Lake Chad basin.

SN	Station	Latitude	Longitude	Data period
Meteorological stations				
1	Abeche	13.85	20.85	1985–2015
2	Banda/MARO	08.40	18.78	1950–2013
3	Bossangoa	06.48	17.43	1950–2013
4	Bongor	10.27	15.40	1980–2015
5	Doba	08.65	16.85	1950–2013
6	Moundou	08.57	16.08	1985–2015
7	N'Djamena	12.13	15.03	1951–2014
8	Sarh	09.15	18.38	1985–2013
9	Yagoua/SAMRY-I	10.37	15.23	1950–2013
10	Sategui Derressia	09.52	16.25	1950–2013
11	Mayo-Tsanaga	10.41	13.82	1950–2013
Hydrometric Stations				
1	N'Djamena	12.12	15.03	1951–2007
2	Sarh	09.15	18.42	1997–2007
4	Bongor	10.27	15.42	1997–2007

**Table 2:** Basic information about the data folders and data files available with the present data article.

Data folder	No. of data file (Excel)	Data file (Excel) Name	Variable	Unit	Data period
Chari-Logone River basin	2	Monthly CRU Climatic data	Max temperature, Min temperature and Rainfall	˚C, mm	1951–2015
		Daily Generated Climatic data	Max temperature, Min temperature and Rainfall	˚C, mm	1951–2015
Komadugu-Yobe River basin	1	Monthly CRU Climatic data	Mean temperature and Rainfall	˚C, mm	1951–2015
YENG basin	1	Monthly CRU Climatic data	Mean temperature and Rainfall	˚C, mm	1951–2015
Lake Fitri basin	1	Monthly CRU Climatic data	Mean temperature and Rainfall	˚C, mm	1951–2015
Streamflow data	2	Daily Simulated natural streamflow data	Streamflow	m^3^/s	1951–2015
		Monthly Observed Streamflow data	Streamflow	m^3^/s	Table 1
Observed Rainfall data	1	Observed station rainfall data	Rainfall	mm	Table 1

YENG refers to the Yedseram, El-beid, Ngadda, and Gubio River basins.

## 2 Experimental design, materials, and methods

Observed hydro-climatic data was obtained from the LCBC as shown in Fig. 1 and Table 1. Since the collected observed climate data was very sparse and not enough for a good climatic and hydrologic study, monthly gridded climatic data (NetCDF format) was extracted from the latest product of CRU (CRU-TS4.00) [2], which is freely available at https://crudata.uea.ac.uk/cru/data/hrg/.

**Fig. 2:**
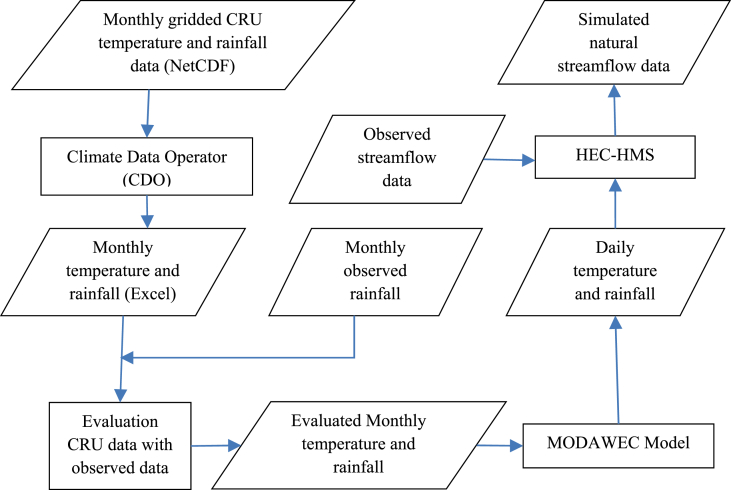
Flowchart diagram to show the material and method used to prepare the data.

First, we divided the Chari-Logone basin into 37 subbasins, Komadugu-Yobe basin into 15 subbasins, YENG basin into 17, and Lake Fitri basin into 12 using Digital Elevation Model data (Fig. 1) in HEC-GeoHMS, which is an extension of ArcGIS. Since Hydrological Modelling system (HEC-HMS) in semi-distributed form needs point data, climatic data (i.e., mean temperature, maximum temperature, minimum temperature, rainfall) was extracted for each subbasin by taking the average of the values of the CRU-grids located inside the corresponding subbasin. Thirty-seven time series were extracted for the Chari-Logone basin, 15 for the Komadugu-Yobe basin, 17 for the YENG, and 12 for Lake Fitri basin. So a total of 81 time series in the study area were prepared for each climatic variable. The CRU climatic data was compared with observed climate data at different sites using some statistical indicators (e.g., correlation and root mean square error) for the evaluation purpose in Mahmood and Jia [1], which showed a good agreement with observed data, with more than 98% correlation. Since HEC-HMS requires daily meteorological inputs for streamflow simulation, the CRU monthly time series (maximum temperature, minimum temperature, and rainfall) were transformed into daily time series using MODAWEC model [3] but only for the Chari-Logone River basin. Because the Chari-Logone River is the main river in the LCB, and it contributes more than 90% of the inflow of the lake [4]. The mathematical explanation of MODAWEC can be found in Liu et al. [3]. At the end, HEC-HMS was successfully calibrated and validated using daily converted CRU data and observed streamflow data at N'Djamena gauge for the period of 1956–1965, and then validated for the periods of 1951–1955 and 1966–1971. The calibration and validation results can be found in Mahmood and Jia [1]. After satisfactory model performance, the natural streamflow was simulated by HEC-HMS for the period of 1951–2015 using daily CRU climatic data. The material and methods used to produce the dataset for the LCB is describe in Fig. 2, using a flowchart.
