# Supplementary material for: Observed and simulated hydro-climatic data for the lake Chad basin, Africa
Source: Data Brief. 2019 May 23;25:104043. doi: 10.1016/j.dib.2019.104043 (PMC6595281; doi:10.1016/j.dib.2019.104043)
Supplement: Multimedia component 1 [file mmc1.doc]

Conflict of Interest and Authorship Conformation Form

Please check the following as appropriate:

- All authors have participated in (a) conception and design, or analysis and interpretation of the data; (b) drafting the article or revising it critically for important intellectual content; and (c) approval of the final version.
- This manuscript has not been submitted to, nor is under review at, another journal or other publishing venue.
- The following authors proclaim that they do not have any conﬂict of interest.:

Author’s name Affiliation

Rashid Mahmood *Key Laboratory of Water Cycle and Related Land Surface*

*Processes/Institute of Geographic Science and Natural Resources R*

*Research, Chinese Academy of Sciences, Beijing 100101, China*

Shaofeng Jia *Key Laboratory of Water Cycle and Related Land Surface*

*Processes/Institute of Geographic Science and Natural Resources R*

*Research, Chinese Academy of Sciences, Beijing 100101, China*
